# Co-ingestion of Antioxidant Drinks With an Unhealthy Challenge Meal Fails to Prevent Post-prandial Endothelial Dysfunction: An Open-Label, Crossover Study in Older Overweight Volunteers

**DOI:** 10.3389/fphys.2019.01293

**Published:** 2019-10-11

**Authors:** David J. Muggeridge, Katarzyna Goszcz, Andrew Treweeke, Janet Adamson, Kirsty Hickson, Daniel Crabtree, Ian L. Megson

**Affiliations:** ^1^Free Radical Research Facility, Division of Biomedical Sciences, Institute of Health Research and Innovation, University of the Highlands and Islands, Inverness, United Kingdom; ^2^Active Health Exercise Laboratory, Division of Biomedical Sciences, Institute of Health Research and Innovation, University of the Highlands and Islands, Inverness, United Kingdom

**Keywords:** antioxidants, polyphenols, red wine, green tea, orange juice, endothelial function, flow-mediated dilatation

## Abstract

Eating a high calorie meal is known to induce endothelial dysfunction and it is reported that consuming drinks rich in antioxidants may be protective against this. In this study we assessed the effects of three antioxidant drinks with considerable disparity in their antioxidant content on endothelial function. Seven apparently healthy overweight and older adults (BMI 25–35; mean age 57 ± 3 years; one male, six females) completed four trials in a randomized counterbalanced design. Water (control), orange juice, green tea, or red wine were consumed with a high calorie meal (>900 kcal). Endothelial function was measured by flow-mediated dilatation immediately before (fasted, baseline) and 2 h after the meal. Blood samples were also obtained for lipid and glucose analysis, plasma nitrite (${\text{NO}}_{2}^{-}$) and oxidized low-density lipoprotein (ox-LDL). Participants returned after a minimum 3 days washout to complete the remaining arms of the study. The results found that the high calorie meal induced a substantial increase in triglycerides, but not cholesterol or glucose, at 2 h after meal ingestion. FMD was significantly reduced by ∼35% at this timepoint, but the effect was not attenuated by co-ingestion of any of the antioxidant drinks. Reduced FMD was mirrored by a reduction in ${\text{NO}}_{2}^{-}$, but ox-LDL was not increased at 2 h after the meal. None of the undertaken measures were influenced by the antioxidant drinks. We conclude that co-ingestion of none of our test antioxidant drinks protected against the substantial post-prandial endothelial dysfunction induced by an unhealthy meal challenge in our sample population at a 2 h timepoint.

## Introduction

Cardiovascular disease (CVD), is the leading cause of morbidity and early mortality (World Health Organization, 2017), accounting for 30% of deaths worldwide (Mendis et al., 2011). Atherosclerosis presents as the primary pathophysiological driver for many CVD manifestations and endothelial dysfunction is a common risk factor associated with the development of atherosclerosis (Kannel, 1996; Schachinger et al., 2000). Diets rich in saturated fatty acids, red meat, sugar, and salt contribute to cardiovascular impairment; 72% of CVD-related deaths are associated with poor diet (Bowen et al., 2018). Excessive nutrient metabolism increases intracellular reactive oxygen species production (Aviram et al., 2004) and leads to impairment of cell function (Wellen and Thompson, 2010). For example, previous human volunteer studies have shown that endothelial function is consistently impaired post-prandial to an unhealthy (high calorie, high fat) challenge meal (de Koning and Rabelink, 2002). Conversely, evidence suggests that the consumption of antioxidant-rich foods (e.g., fruit, vegetables, and whole grains), or moderate red wine consumption promotes healthy endothelial function and lowers the incidence of CVD (Lippi et al., 2010; Opie, 2014).

In the healthy endothelium, nitric oxide (NO) synthesis causes dilatation in response to sheer stress (Furchgott and Zawadzki, 1980; Palmer et al., 1987; Radomski et al., 1987). NO is a powerful anti-atherothrombotic agent (Le Brocq et al., 2008) and loss of functional NO through dysfunctional synthesis or rapid reaction with oxygen-centered free radicals (e.g., superoxide) is understood to be a key early event in atherosclerosis. As well as attenuation of the beneficial effects of NO, reaction of NO with superoxide generates peroxynitrite (ONOO^–^), a cytotoxic species capable of oxidative damage to proteins, lipids, and DNA (Beckman and Koppenol, 1996) that can also cause a down-regulation of endothelial NO synthase (eNOS) activity (Pacher et al., 2007).

Antioxidants constitute a heterogeneous group of substances that share the common property of scavenging reactive oxygen species. Each antioxidant has a unique combination of chemical and biological properties that combine to determine their bioavailability, distribution, mechanism of action, and specificity for the different reactive oxygen species. Diet-derived antioxidants present as highly complex mixtures in food and drink, with the potential for additive or synergistic activity of the individual antioxidants contained therein. Epidemiological evidence gives credence to the hypothesis that populations whose diets are high in antioxidants have lower than expected incidence of CVD. Among the most popular exponents of this hypothesis are the association of low CVD in countries that adopt “Mediterranean diets” (Dontas et al., 2007; Salas-Salvado et al., 2019) and the so-called French paradox, in which consumption of red wine has been proposed as a possible explanation for the surprisingly clear disconnect between high fat intake and low CVD in the French population (Ferrières, 2004). Despite convincing epidemiological evidence in support of the benefits of dietary antioxidants in preventing CVD, it is important to note that a meta-analysis of well-controlled interventional antioxidant supplement studies failed to find benefit in CVD-mediated mortality or morbidity (Bjelakovic et al., 2012).

The key role that oxidative stress plays in endothelial dysfunction makes this a logical place to assess the acute impact of different dietary antioxidant cocktails, particularly in the context of endothelial dysfunction induced by an unhealthy meal. To this end, previous studies have found that some beverages which are high in polyphenols are able to suppress an impairment in endothelial function (Title et al., 2000; Beckman et al., 2001), but it is unclear if this is a generic finding applicable to all drinks high in antioxidants, or specific to those tested. Additionally, trials are often tightly controlled and may not reflect regular lifestyle, or health messages are often misconstrued or simplified when disseminated to the general population. As a result the effects may not be reprodued out with a well controlled environment (e.g., poor ecological validity). In this study we tested the hypothesis that common “antioxidant” drinks taken with an unhealthy breakfast meal protect against endothelial dysfunction in the immediate 2 h, at its peak (Vogel et al., 1997) aftermath of the meal. We were interested in the effects applied to a healthy but at-risk cohort of older and overweight participants following their normal daily dietary habits and were provided with breakfast foods that may regularly be chosen (a continental style breakfast) and a single glass of each of the common “antioxidant” drinks. The primary outcome measure for the study was flow-mediated dilatation (FMD), by way of an indicator of endothelial (dys)function, but we also measured a surrogate marker of constitutive NO generation (plasma nitrite), plasma antioxidant capacity, and plasma ox-LDL in order to help further interpretation of the outcomes of the study. The antioxidant drinks were deliberately chosen for their disparity in antioxidant content and familiarity to participants (e.g., regularly consumed by the population). The drinks chosen were: orange juice – which is rich in vitamin C (Zerdin et al., 2003), green tea – which contains predominantly catechins (Henning et al., 2003), and red wine – which contains alcohol and flavonoids (e.g., resveratrol, delphinidin) (Garrido and Borges, 2013).

## Materials and Methods

### Ethical Approval and Trial Registration

Ethical approval was provided by the University Ethics Committee at the University of the Highlands and Islands (OLETHSHE1376). All procedures described were conducted in accordance with the Declaration of Helsinki 1974 and its later amendments. Informed consent was from all volunteers prior to entering the study and participants could withdraw at any point. This study is registered with ClinicalTrials.gov (NCT03806829).

### Participants

Eight overweight but otherwise apparently healthy older adults (six female) volunteered and provided written informed consent prior to participating in the study. The recruited participants met the following inclusion/exclusion criteria for participation in the study:

– Females were postmenopausal and were not on hormone replacement therapy.
– Had a body mass index (BMI) >25 and <35 kg/m^2^.
– Were non-smokers.
– Reported no history of CVD.
– Reported no history of diabetes (Type 1 or Type 2).
– Were not vegan or with food allergies or intolerances.
– Were not taking any antioxidants over the counter (e.g., vitamins C, E, or polyphenol supplements).
– Did not have a highly active lifestyle.
– Were not taking prescription medication.

A power calculation was conducted, using the variance reported in a previous study investigating the impact of a high fat meal on FMD measured at 2 h (among other time points; Djousse et al., 1999). The power calculation indicated that our crossover study would require eight participants to have 80% power of showing a 1.5% meal-induced reduction in brachial artery diameter at *P* < 0.05 confidence level.

### Experimental Design

The study consisted of four experimental arms in a randomized counterbalanced, crossover design. On experimental days, participants reported to the Centre for Health Science, Inverness following an overnight fast (∼12 h) and in a euhydrated state. Prior to each arm of the study, participants were instructed to continue their normal dietary and lifestyle habits, but were asked to avoid strenuous exercise for 24 h and caffeine for 12 h.

### Procedures

Upon arrival for visit 1, standard anthropometric measures (height and body mass) were assessed prior to completion of the remaining experimental procedures; these initial measures were not assessed at visits 2–4. Following a 10 min rest period, a venous blood sample was collected via venepuncture of a superficial vein of each participant’s arm. Participants then lay supine for 10 min prior to measures of blood pressure (BP) in duplicate, and assessment of FMD of the brachial artery. Participants were then provided with a high calorie, high fat meal (>900 kcal, 50 g fat), and one of four 250 ml experimental drinks [water (control), orange juice, green tea, and red wine] that were to be consumed concomitantly. The typical nutritional composition of the drinks tested can be found in the section containing Supplementary Materials (Table 1). Upon completion of the meal, participants remained seated and inactive for a period of 2 h. Following the 2-h absorption period, blood samples, BP measures, and assessment of brachial FMD were repeated. Subsequent to a minimum of a 3-day washout period between each trial, participants returned to the test centre to complete the remaining three arms of the trial. Following completion of the study, stored samples and FMD video files were analyzed as described in detail below.

**TABLE 1:** Composition of meal choice for the participants.

**Meal**	**Items**	**Weight (g)**	**Calories (kcal)**	**Fat (g)**	**Sugar (g)**
Savory	Croissants × 2	120	447.6	23.6	7.82
	Raspberry or black cherry yogurt	150	169	7.4	19.35
	Butter	20	149	16.5	0.14
	Camembert cheese	40	116	9.1	0.20
	Parma ham	47	36.38	1.1	0.24
Total		337	918	58	27.73
Vegetarian	Croissants × 2	120	447.6	23.6	7.82
	Raspberry or black cherry yogurt	150	169	7.4	19.35
	Butter	20	149	16.5	0.14
	Jam	20	52	0	11.80
	Clotted cream	15	88	9.5	0.35
Total		325	906	57	39.46

### Specific Procedures

#### Flow-Mediated Dilatation

Endothelium-dependent dilatation of the brachial artery was assessed by high-resolution ultrasound imaging and automated vessel-diameter measurements. Ultrasound images were recorded using a Philips Affinity 70 ultrasound machine (Koninklijke Philips N.V., Amsterdam, Netherlands) with a L12 12 MHz linear array transducer. A straight, non-branching segment of the brachial artery above the antecubital fossa was identified in each participant and imaged in a longitudinal plane, with simultaneous capture of blood flow gated pulse wave Doppler imaging. The Doppler gate was set to encompass the majority of the width of the artery, and was angle corrected at 60° or less. Depth, gain, and zoom settings were adjusted to optimize image quality, and recorded for future image acquisition.

Baseline imaging of the brachial artery was recorded for 1 min (*D*_base_) following which, a cuff was inflated to suprasystolic pressure (>220 mmHg) using a rapid cuff inflator (Hokanson, Bellevue, WA, United States) on the upper forearm, distal to the imaging site for 5 min. The cuff was then rapidly deflated and an ultrasound recording from the same segment of the brachial artery was made continuously for a further 4 min to capture peak vessel diameter (*D*_peak_). Brachial artery diameter was measured offline by an automatic edge-detection system (Brachial Analyzer V6, Medical Imaging Applications LLC, Coralville, IA, United States). Change in vessel diameter was calculated using 3 s averaging. Prior to analysis, the data were assessed for the slope of the regression between the logarithmically transformed values of *D*_base_ and *D*_peak_. FMD was expressed as percentage change from *D*_base_ to *D*_peak_, as per the procedures of Atkinson et al. (2013). The slope of the regression line was 0.9 [95% confidence interval (CI) 0.87–0.94].

#### Blood Pressure

Supine measurements of BP were recorded in duplicate by standard auscultation using an automated device (Orman M6, Intelli-Sense, Hoofddorp, Netherlands). Mean arterial pressure (MAP) was calculated using the following equation:

$$\text{MAP}=\frac{\left(2\times \mathrm{diastolic}\,\mathrm{BP}+\mathrm{systolic}\,\mathrm{BP}\right)}{3}.$$

#### Meal Composition

Participants were provided with a choice of two breakfast meals that had similar nutritional value in order to induce an acute decline in endothelial function. The meals were designed to offer a vegetarian and non-vegetarian option and deliver >900 kcal, while being identifiable by the participants (e.g., a typical continental style breakfast). Full details of the meal are included in Table 1.

#### Test Drink Sourcing, Preparation, and Antioxidant Content (Ascorbic Acid Content and Antioxidant Capacity)

Green tea, orange juice, and red wine (Cabernet Sauvignon) were obtained from a local supermarket. Water was sourced from an in-house watercooler. Orange juice and red wine samples were taken from newly open containers. Green tea was brewed prior to analysis by immersing a single bag in 250 ml of hot water (70°C), for 3 min. All samples were equilibrated to room temperature and centrifuged at 5000 × *g*, for 5 min in order to remove any particulates. Collected supernatants were diluted 1:5 in water and 1:100 in assay diluent for ascorbic acid and Oxygen Radical antioxidant Capacity (ORAC) assays, respectively, to make sure all readings were within the range of the standards.

Ascorbic acid content and antioxidant capacity of tested drinks were assessed using an Ascorbic Acid Assay kit (BioVision, Milpitas, CA, United States) and OxiSelect^TM^ ORAC (Cell Biolabs, Inc., San Diego, CA, United States), respectively. In each case, the manufacturer’s protocols were followed without modification. All samples were analyzed in triplicate.

#### Blood Measures (Glucose, oxLDL, Triglycerides, Cholesterol, Plasma Nitrite, Ascorbic Acid, and Antioxidant Capacity)

Blood samples were collected by venepuncture into heparin containing tubes. Glucose levels were assessed in whole blood, immediately after sample collection using a OneTouch Verio Flex glucometer (LifeScan Europe, Zug, Switzerland).

Plasma was isolated by centrifugation of whole blood (1000 × *g*, 4°C, 10 min) and stored (−80°C), unless otherwise stated. Oxidized LDL was measured using an oxidized LDL ELISA kit (Mercodia AB, Uppsala, Sweden), and triglyceride concentrations were assessed by colorimetric assay (Cayman Chemical, Ann Arbor, MI, United States) following the manufacturer’s guidelines. All samples were analyzed in duplicate.

The total cholesterol assay was performed according to manufacturer’s protocol (Abcam, Cambridge, United Kingdom), with minor modifications. Briefly, after centrifugation, plasma samples were aliquoted and stored at −152°C. For measurement of total cholesterol (HDL and LDL/VLDL) plasma samples were diluted 1:20 in assay buffer. Plasma HDL and LDL/VDL fractions were separated using supplied precipitation buffer. Both fractions were further diluted 1:1 in phosphate-buffered saline (PBS), to ensure all samples were within the range of the standards. All samples were analyzed in duplicate. eHDL was determined using the cholesterol assay above. Whereas eLDL was estimated using Friedewald equation presented below (Martin et al., 2013):

eLDL=TC⁢(total⁢cholesterol)-eHLD⁢(total⁢HDL)-(TG⁢(triglycerides)/5).

This equation uses a fixed ratio of triglyceride levels to very low lipoprotein (VLDL) cholesterol of 5:1 (Martin et al., 2013).

Plasma nitrite was analyzed by chemiluminescence according to the manufacturer’s protocol. Sodium iodide and glacial acetic acid were placed in a glass purge vessel at room temperature and connected to the NO analyzer (Sievers NOA 280i, Analytix, United Kingdom). The vessel was purged with nitrogen gas. A standard curve was created by injecting 100 μL of nitrite solutions at concentrations up to 1000 nM. Plasma samples were thawed and 100 μL of the thawed sample was injected immediately into the purge vessel. The nitrite content of the plasma was calculated from the area under the curve using Origin software (version 7.1). All samples were analyzed in duplicate.

Plasma antioxidant capacity was assessed by the same ORAC assay as mentioned previously. Prior to analysis, plasma samples were diluted 1:200 in assay diluent. Ascorbic acid content in plasma samples was determined using an Ascorbic Acid kit (FRASC) (Cell Biolabs, Inc., San Diego, CA, United States) following the manufacturer’s instructions. All samples were analyzed in duplicate.

### Statistical Analysis

The distribution of data collected was assessed using Shapiro–Wilk normality tests prior to data analysis. Two-factor repeated measures analysis of variance (ANOVA) for “time” (pre- and 2 h post-challenge meal), “condition” [water (control), orange juice, green tea, and red wine], and their interaction was used to assess differences in blood parameters and FMD. To evaluate the influence of *D*_base_ on FMD, we used a linear mixed model with allometric scaling of differences in FMD. The dependent variable was “*D*_difflogs_,” fixed factors were condition and time and the log of *D*_base_ was the covariate used in the analysis (Atkinson et al., 2013). Delta changes between conditions were also analyzed by a one-way ANOVA. Bonferroni *post hoc* correction was applied when assessing multiple comparisons. Wilcoxon-Rank and Kruskal–Wallis tests were used for non-normally distributed data. All statistical analyses were performed using GraphPad Prism version 6 (GraphPad Software Inc., San Diego, CA, United States). Data are presented as mean ± standard deviation (SD) unless otherwise stated. Mean difference, 95% CIs, and effect size (Cohen’s *d*) are included where appropriate. *P* < 0.05 was considered to indicate a significant difference between means.

## Results

### Recruitment

Twenty-one potential participants registered interest in the study, of whom eight met the inclusion/exclusion criteria. One participant subsequently dropped out of the study due to ill health; the remaining seven participants (six female) were randomized for drink order using an on-line randomization tool^1^ and were scheduled for four visits to the facility as per the experimental design. The participant characteristics are summarized in Table 2.

**TABLE 2:** Participant baseline characteristics.

**Characteristic/baseline measurement**	**Value**
Age (years)	57 ± 3
Height (cm)	166 ± 10
Weight (kg)	83.6 ± 11.3
Body mass index (kg/m^2^)	30.5 ± 5.1
Systolic blood pressure (mmHg)	127 ± 14
Diastolic blood pressure (mmHg)	83 ± 8
Mean arterial pressure (mmHg)	97 ± 10

*Data displayed as mean ± SD.*

### Antioxidant Capacity of Test Drinks Prior to Consumption

**FIGURE 1:**
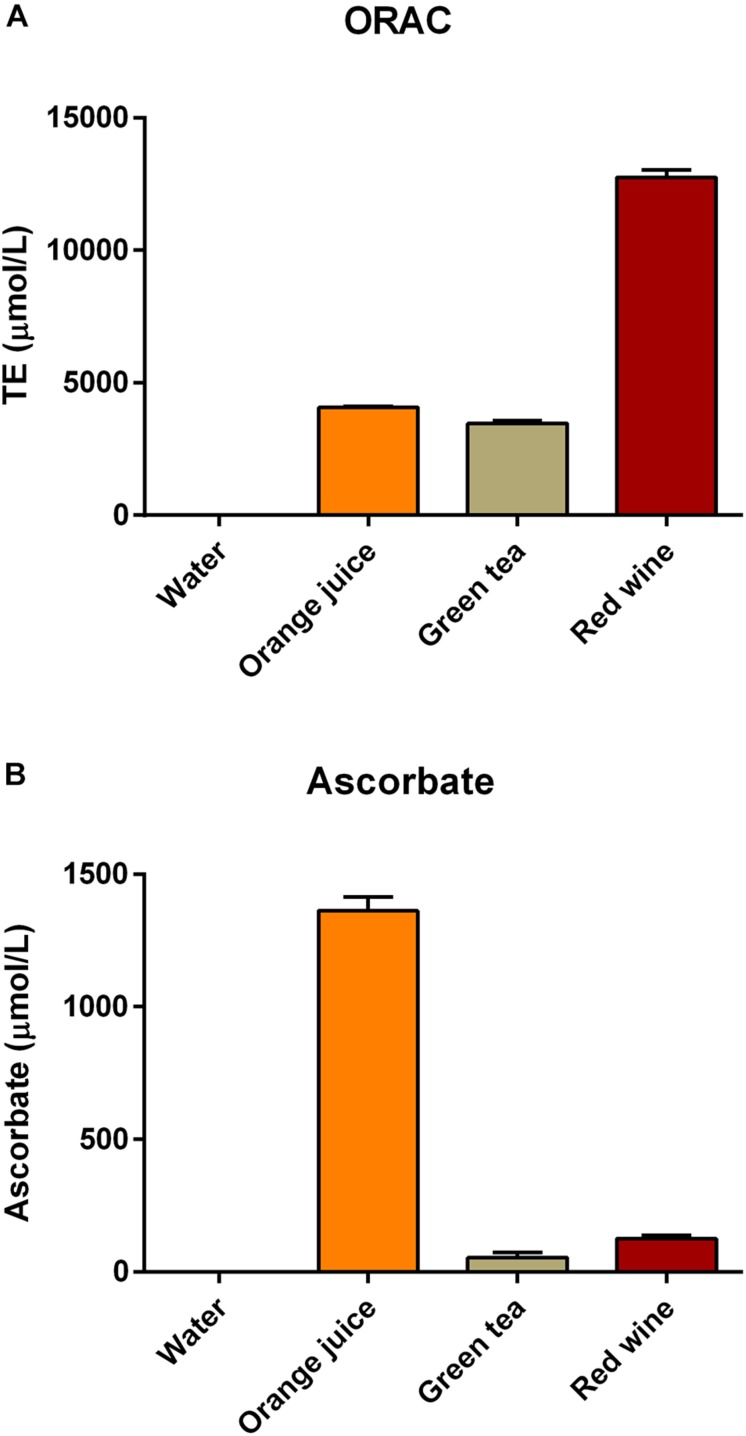
Antioxidant capacity of the drinks used in this study [measured using **(A)** the ORAC assay and **(B)** the FRASC assay for ascorbate].

Samples of drinks were analyzed for total antioxidant capacity prior to consumption using the ORAC assay. Water was found not to have any detectable antioxidant capacity, orange juice (TE = 4073 ± 55 μM) and green tea (TE = 3471 ± 180 μM) had similar antioxidant capacities, while the selected red wine (TE = 12752 ± 489 μM) had antioxidant capacity approximately threefold higher than the other two drinks (Figure 1A). Water did not have any detectable ascorbate, but all of the other test drinks contained ascorbate (orange juice 1361.7 ± 90.9 μM; green tea 54.9 ± 34.4 μM; red wine 125.7 ± 21 μM; Figure 1B). The ascorbate level was substantially higher in the orange juice than any of the other test drinks.

### Effect of Meal Challenge on Post-prandial (2 h) Plasma Glucose, Triglycerides, Cholesterol, and Ascorbate Measures

**FIGURE 2:**
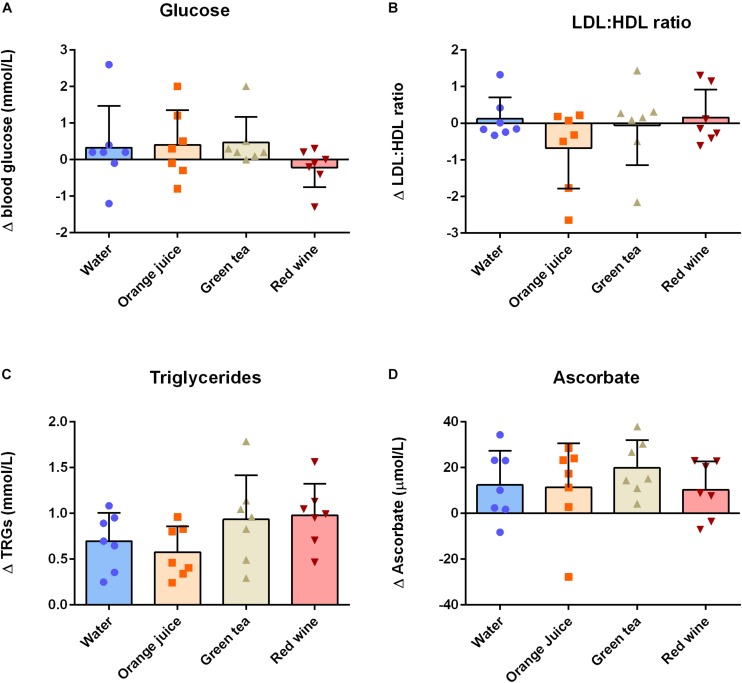
Change (Δ) between baseline and 2 h post-prandial measures of **(A)** plasma glucose, **(B)** plasma eLDL:eHDL ratio, **(C)** plasma triglycerides, and **(D)** plasma ascorbate (*n* = 7). Triglycerides and ascorbate concentrations were significantly increased at 2 h post-prandial, but there was no effect of any of the antioxidant drinks on these measures (*P* > 0.05). Data displayed as individual values (data points), group mean (filled bars), and standard deviation (error bars).

There were no main effects of “time” or “condition” or their interaction on plasma glucose (Figure 2A and Table 3; *P* > 0.05), plasma total cholesterol, HDL, eLDL (Table 3; all *P* > 0.05), or LDL/HDL ratio (Figure 2B and Table 3; *P* > 0.05). There was a main effect of “time” on plasma triglyceride concentration (Figure 2C and Table 3; *P* < 0.001). *Post hoc* analysis revealed that plasma triglycerides were elevated 2 h after the meal challenge in all conditions (Table 3; water: *P* = 0.001, mean difference = −0.696, 95% CI −1.064 to −0.326, orange juice: *P* = 0.0013, mean difference = −0.576, 96% CI −0.945 to −0.207, green tea: *P* < 0.001, mean difference = −0.934, 95% CI −1.303 to −0.565, red wine: *P* < 0.001, mean difference = −0.978, 95% CI −1.347 to −0.609). The change in plasma triglycerides was not significantly different between conditions (Figure 2C; *P* > 0.05). Plasma ascorbate concentrations were increased in all four groups, but there was no significant difference between the test drinks and the water control (Figure 2D and Table 3; *P* > 0.05 for all).

**TABLE 3:** Group data (*n* = 7) of outcome measures for each drink condition pre- and 2 h post-challenge meal.

**Parameter**	**Water (control)**		**Orange juice**		**Green tea**		**Red wine**	
	**Pre**	**Post**	**Pre**	**Post**	**Pre**	**Post**	**Pre**	**Post**
Glucose (mmol/L)	5.0 ± 0.4	5.4 ± 1.3	5.0 ± 0.2	5.4 ± 1.0	5.2 ± 0.5	5.7 ± 1.1	5.0 ± 0.5	4.8 ± 0.4
Triglycerides (mmol/L)	14.3 ± 7.7	26.5 ± 10.6^a^	20.1 ± 12.2	32.7 ± 15.5^a^	16.6 ± 7.1	33.0 ± 14.1^a^	16.7 ± 10.6	32.6 ± 15.8^a^
Total cholesterol (mmol/L)	6.8 ± 1.4	6.8 ± 1.6	7.1 ± 1.8	6.4 ± 1.4	7.0 ± 1.9	6.8 ± 2.7	6.6 ± 2.3	6.9 ± 2.1
HDL-C (mmol/L)	1.7 ± 0.6	1.7 ± 0.7	1.9 ± 1.0	1.9 ± 1.1	1.9 ± 0.8	1.9 ± 0.8	1.8 ± 0.8	1.8 ± 0.9
LDL-C (mmol/L)	4.9 ± 1.2	4.8 ± 1.4	5.0 ± 2.0	4.2 ± 1.2	4.9 ± 1.6	4.6 ± 2.6	4.6 ± 2.2	4.7 ± 2.1
Oxidized LDL (U/L)	69.0 ± 13.9	66.2 ± 18.4	66.4 ± 10.6	68.4 ± 13.1	68.6 ± 20.4	65.8 ± 16.4	65.6 ± 14.4	63.6 ± 16.9
Plasma ORAC (TE; mmol/L)	9.7 ± 0.8	9.8 ± 1.7	10.4 ± 1.0	9.8 ± 1.2	10.2 ± 1.4	10.1 ± 1.6	10.5 ± 0.9	10.7 ± 0.9
Plasma ascorbate (μmol/L)	67.3 ± 10.8	79.7 ± 11.0^a^	67.8 ± 15.6	79.1 ± 7.6^a^	60.5 ± 6.6	80.5 ± 9.6^a^	64.5 ± 6.8	74.9 ± 9.0^a^
Plasma nitrite (nM)	45 ± 18	38 ± 11	32 ± 14	33 ± 30	32 ± 13	28 ± 11	39 ± 14	34 ± 14
FMD (%)	6.4 ± 2.9	4.5 ± 2.6	6.7 ± 2.8	3.8 ± 3.4	6.1 ± 3.8	4.3 ± 2.6	6.8 ± 4.2	5.2 ± 3.6
**D*_*base*_* (mm)	4.3 ± 0.8	4.6 ± 0.8	4.3 ± 0.8	4.4 ± 0.9	4.5 ± 1.0	4.5 ± 0.8	4.5 ± 1.0	4.8 ± 1.1

*Data are presented as mean ± SD. HDL-C, high-density lipoprotein cholesterol; LDL-C, low-density lipoprotein cholesterol; ORAC, oxygen radical absorbance capacity; FMD, flow-mediated dilatation; *D*_*base*_, baseline brachial artery diameter. ^*a*^Differences between pre- and post-time points within groups (two-factor ANOVA “time effect”; *P* < 0.05). There was no significant difference between conditions at either timepoint for any of the parameters (Bonferroni post-test).*

### Effect of Meal Challenge on Post-prandial (2 h) Flow-Mediated Dilatation and Plasma Nitrite

**FIGURE 3:**
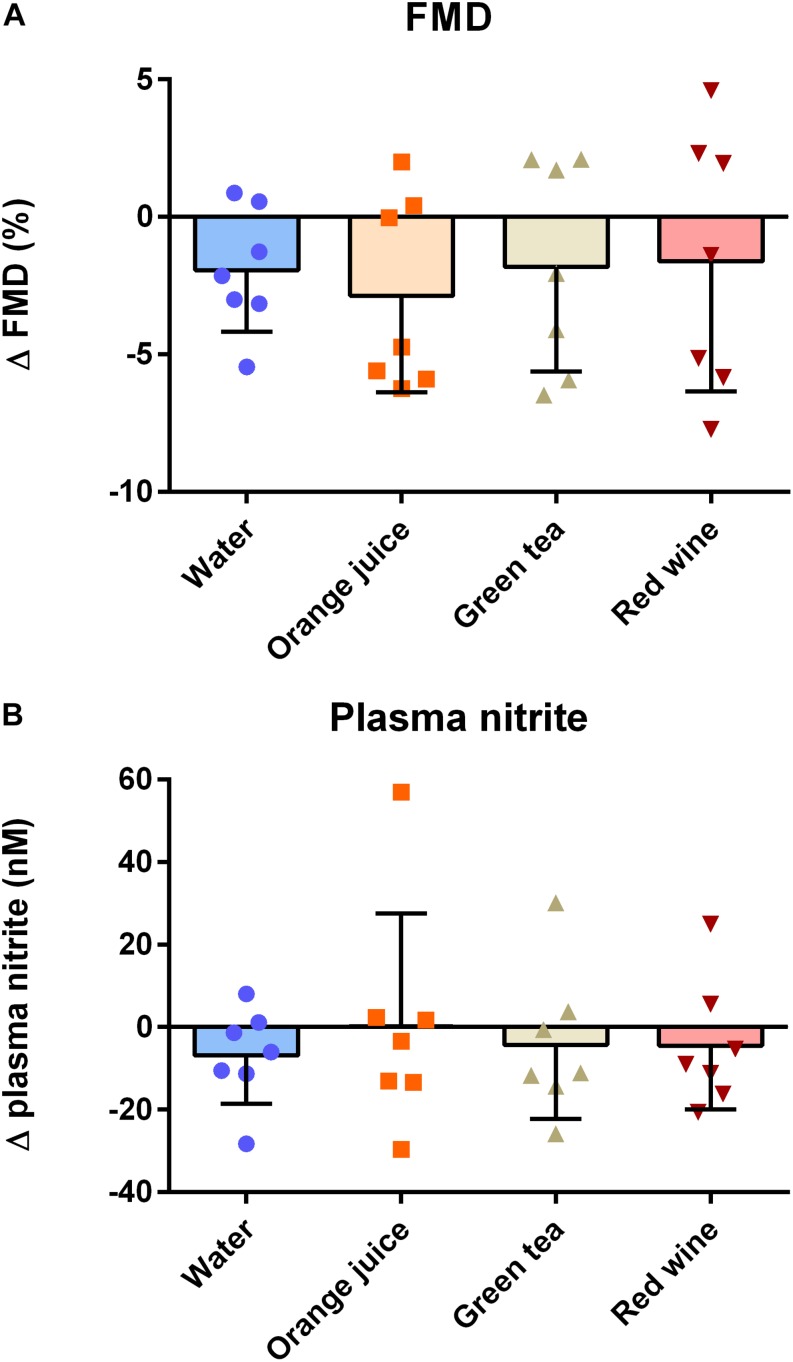
Effect of meal challenge on 2 h post-prandial **(A)** FMD and **(B)** plasma nitrite, as a surrogate of NO. Post-prandial FMD was significantly reduced compared to baseline, but antioxidant drinks had no significant impact on the extent of the effect compared to the water control (*P* > 0.05, one-factor ANOVA). Similarly, plasma nitrite was significantly reduced at the post-prandial timepoint compared to baseline (*P* < 0.05; Kruskal–Wallis), but there was no significant effect of antioxidant drinks on the extent of the effect.

There was a main effect of “time” on FMD pre- to post-meal challenge using both ANOVA (*P* = 0.007) and linear mixed model (*P* = 0.024) analyses. While FMD was reduced 2 h after the meal challenge (mean difference = −2.06%, 95% CI −3.73 to −0.39%, *d* = 0.66), *post hoc* analysis revealed that this did not reach statistical significance in any of the individual conditions (Table 3). Additionally, the change in FMD was not significantly different between conditions (Figure 3A; *P* > 0.05). Similarly, plasma nitrite was significantly lower following the challenge meal (*P* = 0.048, mean difference = 6 nM, 95% CI 0.1–11.3 nM, *d* = 0.21); however, this change was not different between any of the conditions (Figure 3B; *P* = 0.998).

### Effect of Meal Ingestion on Post-prandial (2 h) Markers of Plasma Antioxidant Capacity and Oxidative Stress

**FIGURE 4:**
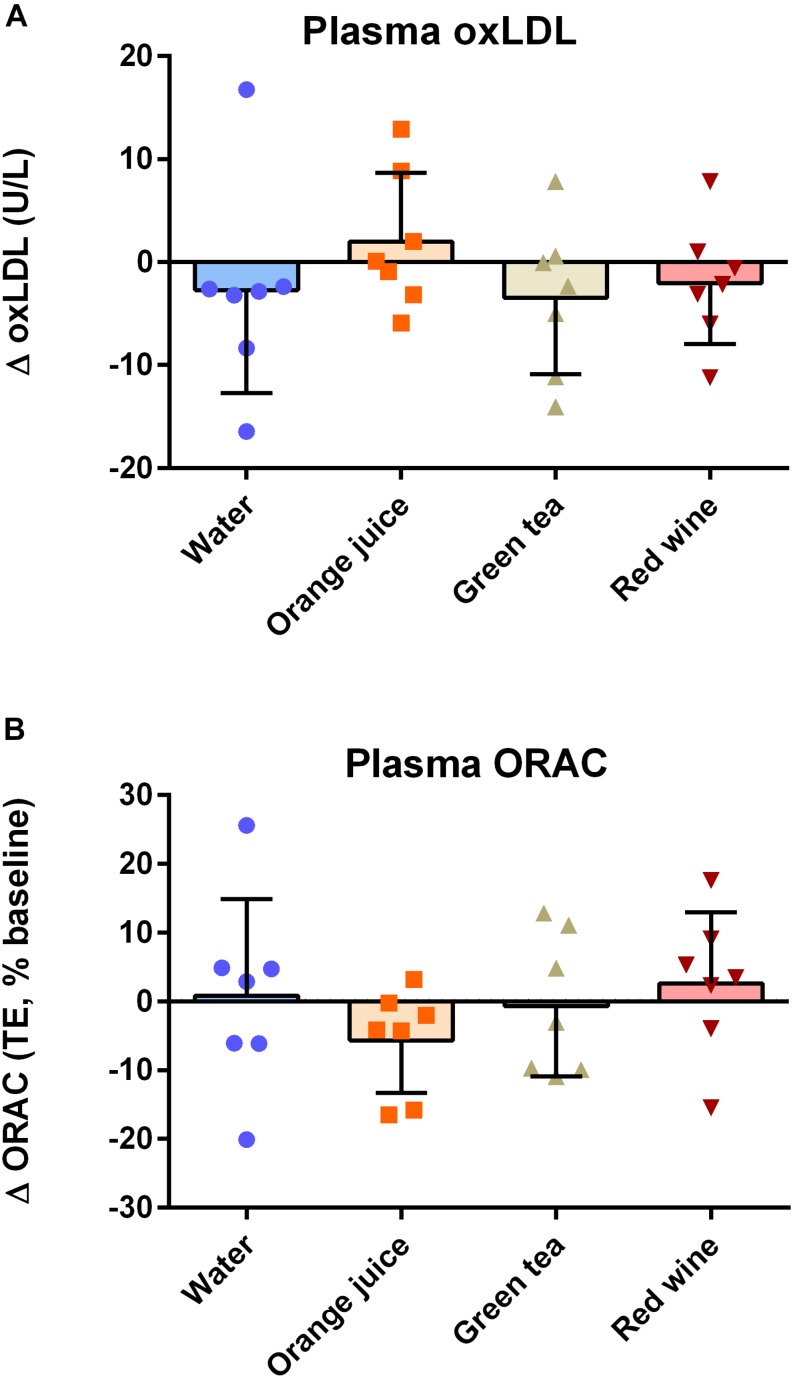
Effect of meal challenge on 2 h post-prandial **(A)** ox-LDL **(B)** plasma antioxidant capacity (ORAC). There was no significant difference between plasma ox-LDL or ORAC values in any of the groups (*P* > 0.05).

There were no effects of “time,” “condition,” or their interaction on ox-LDL (Figure 4A; *P* > 0.05) or plasma ORAC measures (Figure 4B; *P* > 0.05).

## Discussion

An unhealthy meal challenge was characterized by an increase in plasma triglyceride levels and induced a substantial (35%, *d* = 0.66) reduction in FMD across the four arms of the study, but there was no impact of orange juice, green tea, or red wine on this deleterious effect. Plasma nitrite showed a very similar trend, with an overall reduction registered in response to the meal challenge, but no impact of antioxidant drinks at the 2 h post-prandial point. There was no significant effect of the challenge meal on ox-LDL or plasma antioxidant capacity and no impact of antioxidant drinks on these measures either. We infer from the results that the antioxidant test drinks were not able to protect against challenge meal-induced endothelial dysfunction at this timepoint, either because oxidative stress is not a major driver for the effect, or because the temporal peak for antioxidant effects is incongruous with that of endothelial dysfunction (2 h post-ingestion).

### Influence of Meal Challenge on Lipids, Glucose, and Markers of Oxidative Stress

The lipid meal challenge was associated with increases in triglycerides, but not glucose or cholesterol. These findings are consistent with previous reports that showed substantial increase in triglyceride levels post high-fat meal ingestion (Burton-Freeman et al., 2010; Peluso et al., 2014; Henning et al., 2018). Previous studies also noted no significant increase in total cholesterol level at 2 h post-prandial point (Djousse et al., 1999; Peluso et al., 2014). Nevertheless, some of the reports showed later increase in cholesterol level detectable only ≥4 h after consumption of a high-fat meal (Peluso et al., 2014; Liu et al., 2015).

The lack of impact on glucose at 2 h is consistent with a study population without diabetes. The postprandial glucose level in healthy participants typically peaks at ∼30 min post unhealthy meal ingestion and rapidly returns to baseline before 2 h (Phillips et al., 2013).

Evidence suggests that oxidation of low-density lipoprotein (LDL) contributes to the process of atherogenesis and that ox-LDL is a crucial player. Plasma ox-LDL is significantly elevated in patients suffering from acute myocardial infraction and cerebral infraction (Tsuzura et al., 2004; Itabe, 2012). Plasma ox-LDL might have been increased in the face of a load of lipids oxidized in the exogenous environment (e.g., oxidized in the cooking process) or on account of induction of endogenous oxidative stress, leading to oxidation of circulating lipids, already incorporated into LDL particles. That neither is apparent at 2 h might be indicative that neither source of ox-LDL was relevant to this meal challenge. These results are consistent with previous findings that show no increase in ox-LDL concentration shortly after high-fat meal challenge (0–3 h) (Phillips et al., 2013). Interestingly, some other studies reported a small increase in plasma ox-LDL level after high-fat meal consumption. The change, however, has been only observed at a later post-prandial timepoint (≥4 h) (Burton-Freeman et al., 2010; Di Renzo et al., 2017; Henning et al., 2018). This result could indicate that the absorption, synthesis, or clearance of the ox-LDL prevented an increase at 2 h.

This finding in itself is important because ox-LDL is understood to be able to induce endothelial dysfunction in its own right (Kita et al., 2001; Valente et al., 2014); the results of our study might suggest that any effects of the meal challenge on endothelial function are not mediated by circulating ox-LDL and probably do not reflect increased oxidative stress that is reflected in the plasma compartment at this time point. However, others have observed that an increase in ox-LDL can be seen subsequent to endothelial dysfunction, caused by glucose and/or lipid loading, but that the timing suggests that the rise in ox-LDL is an effect rather than cause of dysfunction (de Koning and Rabelink, 2002; Henning et al., 2018).

That the antioxidant drinks had no impact on circulating lipids, glucose, or ox-LDL, intimates that they do not modulate meal digestion or metabolic function, which allows the focus to switch to their impact on antioxidant status. Importantly, none of the antioxidant drinks had any impact on total plasma antioxidant status, as measured by ORAC. In addition, while an increase in ascorbate was registered in all of the study arms, there was no association with the concentration of ascorbate in the test drinks, suggesting that the increase is independent of the drinks and possibly more closely related to the meal challenge itself. These are important observations because they imply that the antioxidant status recorded in the drinks prior to ingestion does not translate to a change in plasma antioxidant status at this timepoint. This finding might be related to the poor absorption of many of the antioxidants involved (e.g., polyphenols from green tea or red wine), rapid metabolism, and clearance or absorption into cells from the plasma compartment. However, it might simply reflect the fact that absorbed antioxidants are insufficient to alter the pre-existing capacity of plasma that is already very high in antioxidants (Goszcz et al., 2017b).

### Endothelial Dysfunction and Unhealthy Meal Challenge

Our findings broadly support those from previous studies in the context of the impact of meal challenge on endothelial function (Vogel et al., 1997; de Koning and Rabelink, 2002; Barringer et al., 2011). The dramatic reduction in endothelial function across all conditions was similar in magnitude (∼2% decrease in FMD) to that seen in a younger cohort of healthy volunteers (Plotnick et al., 1997; Djousse et al., 1999), but constituted a higher percentage change from baseline on account of the lower baseline FMD measurements in our older cohort of individuals (∼35% of baseline). The effect was associated with a reduction in plasma nitrite – a surrogate marker for NO – suggesting that endothelium-derived NO is reduced at 2 h after an unhealthy meal. Whether the loss of nitrite reflects a reduction in synthesis of NO (i.e., downregulation or reduced activation of eNOS) or increased loss of NO through scavenging and reaction that result in alternative products is unknown, but it is reasonable to speculate that reduced functional NO is at least in part responsible for the post-prandial reduction in FMD.

### Co-ingestion of Antioxidant Drinks Fails to Protect Against Endothelial Dysfunction, to Influence Plasma Antioxidant Levels or to Influence Markers of Oxidative Stress

There was no significant impact of co-ingestion of any of the antioxidant drinks on endothelial function or plasma nitrite compared to the control (water) arm of the study. These results are in contradiction to much of the epidemiological data and to findings from longer-term studies with similar antioxidant drinks with regard to overall cardiovascular health (Aviram et al., 2004; Wolfram, 2007; Morand et al., 2011; Aptekmann and Cesar, 2013; Tjelle et al., 2015). However, our study was specifically designed to determine the impact of antioxidant drinks on endothelial dysfunction induced by a challenge meal, based on previous studies that suggest a maximal effect at 2 h after the meal (Vogel et al., 1997). The hypothesis was predicated on the fact that endothelial dysfunction is mediated, at least in part, by oxidative stress (Le Brocq et al., 2008) and that at least some of the antioxidants contained in the drinks would be absorbed sufficiently rapidly, and be present in the correct compartment within 2 h of ingestion. For the hypothesis not to be supported, it is reasonable to predict that at least one of the requirements for antioxidant protection against meal-induced endothelial dysfunction has not been satisfied.

Oxidative stress is a well-known mediator of endothelial function, both via direct inactivation of the protective endothelium-derived anti-atherosclerotic and vasodilator factor, NO, and through cytotoxic effects on endothelial cells in the longer term (Nedeljkovic et al., 2003; Incalza et al., 2018).

That an unhealthy meal might induce endothelial oxidative stress is a reasonable hypothesis, given that cellular metabolism is likely to change in the acute phase, with the potential to drive intracellular reactive oxygen species generation (Tsai et al., 2004). The acute stress of calorie loading might also drive inflammation (Erridge et al., 2007; Herieka and Erridge, 2014), which is also a potential source of harmful free radicals (Closa and Folch-Puy, 2004). However, there are other potential drivers for endothelial dysfunction that are not necessarily mediated by oxidative stress (e.g., advanced glycation end products, hyperinsulinemia, genetic predisposition, hypertension, lipemia, thrombosis) and it is possible that the effect seen are driven by alternative mechanisms to oxidative stress (Sena et al., 2013).

Bioavailability of antioxidants is a major issue facing the field. We know that vitamin C (e.g., in orange juice) is absorbed rapidly and increases plasma vitamin C. However, concentrations do not rise above ∼80 μM on account of active renal clearance at higher concentrations, so the impact on plasma antioxidant capacity in any individual is predicated on their pre-existing vitamin C concentration. Polyphenols, on the other hand, are poorly absorbed and subject to substantial metabolism in the gut and *in vivo* concentrations rarely reach >1 μM in the blood, prompting suggestions that they will not have any significant impact on plasma antioxidant capacity (Nicholson et al., 2010; Goszcz et al., 2017a, b). Instead, there is a growing body of evidence to suggest that polyphenols, or their metabolites, act to stimulate endogenous antioxidant defenses through activation of the NRF-2/ARE-1 signaling pathway (Grossini et al., 2015; Tang et al., 2018). Should the latter be the case, the timeframe for endogenous antioxidant activity is likely to be extended by several hours to allow activation of these complex intracellular processes. Some previous studies have shown that berry-rich juice consumption (a source of polyphenols) reduces markers of postprandial stress (triglycerides, cholesterol, inflammatory markers) a few (>4) hours after ingestion of a high fat meal, but did not report any protective effects toward endothelial function at the acute stage of the study (Burton-Freeman et al., 2010; Peluso et al., 2014). Conversely, a recent study by Istas et al. (2019) observed a 1.4% improvement in FMD following the acute ingestion of aronia berry extract.

Perhaps of particular surprise is that the decline in endothelial function was unchanged during the red wine trial. Alcohol ingestion itself has the potential for a wide range of direct and indirect impacts on vascular function and hydration levels coupled with an antioxidant potential which collectively have previously been shown to affect vascular function directly (Greenberg et al., 1993; Puddey et al., 2001). Nevertheless, in the present study there was no obvious physiological consequence of red wine on vascular function as a whole, let alone endothelial function.

On the basis of this study, it is not possible to speculate on which of the above might explain the lack of effect of the antioxidant drinks on unhealthy meal-induced endothelial dysfunction at 2 h, but ox-LDL does not appear to be responsible. Our data also indicate that there is almost no impact of the antioxidant drinks on plasma antioxidant capacity, despite the high levels found in some of the drinks prior to consumption. It is reasonable to conclude with some certainty that there is insufficient antioxidant accumulation in the blood at 2 h following the consumption of our test drinks to mediate significant direct antioxidant effects in this compartment. Any polyphenol-mediated effects could require the induction of intracellular mechanisms that are likely to develop several hours after ingestion.

### Strengths and Limitations

Particular strengths of the study include the randomized crossover study design, utilizing a homogenous sample group in an ecologically valid testing scenario. We also believe that a particular strength of the study includes the use of FMD as our primary measure of endothelial (dys)function, coupled with an array of secondary measures in circulation (including plasma nitrite) to further explain the induced dysfunction caused by our challenge meal. We acknowledge that limitations to the study include the small sample size with the findings in this study generalizable across the population. A retrospective power calculation using the standardized difference generated from our study indicated that the study had 80% power of detecting a gross (∼75%) antioxidant drink-induced protection against the endothelial dysfunction associated with ingestion of an unhealthy meal. It is acknowledged that a much bigger study would be required to detect a more modest protective effect. Further, the snapshot aspect of a single timepoint in response to the challenge meal and antioxidant intervention drinks is a limitation in the current study. Despite this, previous research suggests that the time point selected correlates with peak endothelial dysfunction and provided an appropriate time point for further investigation (Vogel et al., 1997; Djousse et al., 1999; de Koning and Rabelink, 2002). The small discrepancies in the meal choices may have also had an impact upon our findings and are acknowledged as a limitation in our interpretation of the data collected. Lastly, we did not control dietary intake throughout the duration of data collection for participants. As noted above, we believe that this is a strength of the study design, because it helps create an ecologically valid testing scenario; however, it also represents a limitation because we are unable to assess the impact of long-term diet on our outcome measures. Nevertheless, the overnight (12 h) fast prior to data collection was standardized in order to ensure that we were able to assess the acute impact of the challenge meal combined with antioxidant drinks from a standardized basal endothelial function. Finally, there was an imbalance of males:females in the study. Sex always has the potential to influence outcome of human studies, but our sample size was too small to investigate any clear difference between responses in males and females, given that it was not a specific objective of the study.

## Conclusion

In conclusion, this study confirms that an unhealthy meal induces endothelial dysfunction at 2 h, but that the effect is not modulated in the acute phase by co-ingestion of antioxidant drinks containing either vitamin C (orange juice) or polyphenols (hesperidin in orange juice, catechins in green tea, and delphinidin and resveratrol among others in red wine) within the study population. Further research is required to establish whether an antioxidant-mediated benefit develops in time, or indeed whether meal-induced endothelial dysfunction is independent of oxidative stress altogether. A strong temporal aspect to antioxidant protection would support either pre-emptive dosing in advance of meal challenges, or repeated “dosing” with antioxidant drinks that would be achieved through lifestyle change. Further investigation using a larger sample size and more diverse population of participants is required in order to confirm the findings of this study.

## Data Availability Statement

Datasets are available on request: The raw data supporting the conclusions of this manuscript will be made available by the authors, without undue reservation, to any qualified researcher.

## Ethics Statement

The studies involving human participants were reviewed and approved by the University of the Highlands and Islands, University Ethics Committee. The patients/participants provided their written informed consent to participate in this study.

## Author Contributions

DM and IM performed the study concept and design with input from KH, KG, and DC. DM, KG, AT, JA, and KH carried out the data collection. DM, KG, and IM performed the data analysis. IM prepared the original manuscript draft. DM, KG, AT, JA, KH, DC, and IM reviewed and edited the manuscript. All authors accepted the final submitted version of the manuscript.

## Conflict of Interest

The authors declare that the research was conducted in the absence of any commercial or financial relationships that could be construed as a potential conflict of interest.

## Footnotes

1 www.randomizer.org
