# Supplementary material for: Co-ingestion of Antioxidant Drinks With an Unhealthy Challenge Meal Fails to Prevent Post-prandial Endothelial Dysfunction: An Open-Label, Crossover Study in Older Overweight Volunteers
Source: Front Physiol. 2019 Oct 11;10:1293. doi: 10.3389/fphys.2019.01293 (PMC6797614; doi:10.3389/fphys.2019.01293)
Supplement: Supplementary file 1 [file Table_1.DOCX]

Supplementary Material

# Supplementary Data

Supplementary Material should be uploaded separately on submission. Please include any supplementary data, figures and/or tables. All supplementary files are deposited to FigShare for permanent storage and receive a DOI.

Supplementary material is not typeset so please ensure that all information is clearly presented, the appropriate caption is included in the file and not in the manuscript, and that the style conforms to the rest of the article. To avoid discrepancies between the published article and the supplementary material, please do not add the title, author list, affiliations or correspondence in the supplementary files.

# Supplementary Figures and Tables

For more information on Supplementary Material and for details on the different file types accepted, please see [here](http://home.frontiersin.org/about/author-guidelines#SupplementaryMaterial). Figures, tables, and images will be published under a Creative Commons CC-BY licence and permission must be obtained for use of copyrighted material from other sources (including re-published/adapted/modified/partial figures and images from the internet). It is the responsibility of the authors to acquire the licenses, to follow any citation instructions requested by third-party rights holders, and cover any supplementary charges.

## Supplementary Figures


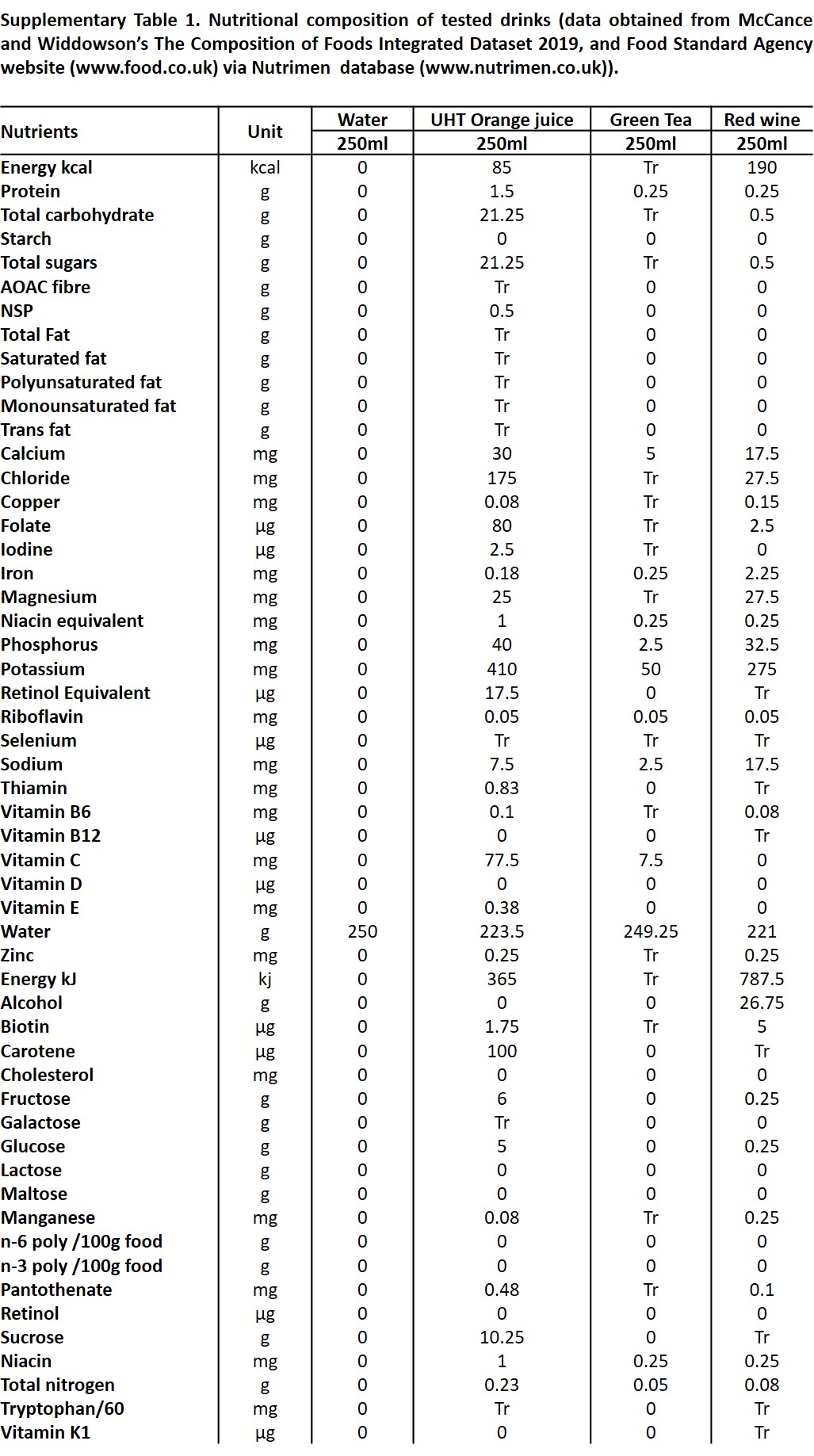


**Supplementary Table 1.** Nutritional composition of tested drinks (data obtained from McCance and Widdowson’s The Composition of Foods Intergrated Dataset 2019 (PHE Publications., 2019), and Food Standard Agency website ([www.food.co.uk](http://www.food.co.uk)) via Nutrimen database (www.nutrimen.co.uk)).

Public Health England. McCance and Widdowson's The composition of foods integrated dataset 2019: 1-41 user guide, *PHE Publications, 2019*
